# Hematological and biochemical indices, growth performance, and puberty of goats fed with Mombasa and blue panic as salt-tolerant alternatives to alfalfa under arid conditions

**DOI:** 10.3389/fvets.2022.961583

**Published:** 2022-10-18

**Authors:** Hany Ahmed Zaher, Ayman Mesalam, Adel Ibrahim Al Bloushi, Ameer Tolba, Ayman A. Swelum, Ihsan Abu-Alrub

**Affiliations:** ^1^Research and Development Division, Abu Dhabi Agriculture and Food Safety Authority, Abu Dhabi, United Arab Emirates; ^2^Department of Theriogenology, Faculty of Veterinary Medicine, Zagazig University, Sharkia, Egypt

**Keywords:** Mombasa, blue panic, goats, growth, hematological indices

## Abstract

The objective of this study was to evaluate the impact of Mombasa or blue panic as a salt-tolerant alternative to alfalfa on growth performance, puberty, blood hematology, serum metabolites, and serum mineral profile in growing goats. Twenty-four growing goats of 4 months old age with 14.45 ± 0.6 kg average body weight were assigned to three treatment diets with 8 animals per treatment. Weights of each animal were measured at the onset of the trial and subsequently on a weekly basis until the end of the trial duration. A pair of blood samples were collected from each goat *via* a jugular vein puncture and were subjected to either hematological or biochemical analysis. The results showed that treatment diets had no significant effects (*P* > 0.05) on the final body weight and total body weight gain. However, blue panic had significantly increased (*P* < 0.05) neutral detergent fiber and crude protein digestibility. The diet-influenced MCV was significantly higher (*P* < 0.05) in the Alfalfa group. The serum concentration of glucose was significantly increased (*P* < 0.05) in the blue panic-fed group, while the urea was increased in the Mombasa-fed group. Additionally, the serum concentrations of P, Na, and Cl were significantly increased (*P* < 0.05) in the blue panic-fed group, but Mombasa significantly increased (*P* < 0.05) the K concentration. In conclusion, the study indicated that blue panic ranked the best among salt-tolerant alternatives to replace alfalfa, resulting in better feed utilization, serum metabolites, and serum minerals with no adverse effects on growth performance and puberty. This study provides new insight into the shift to the cultivation of salt-tolerant plants with a high level of crude protein in arid areas as a potential approach for the sustainability of the livestock industry.

## Introduction

Over the past decades, changes in climatic conditions have resulted in persistent droughts and shortages in animal feed, which threaten animals raised in tropical climates (1, 2). Tropical and subtropical regions are the most affected areas by global warming. In arid and semiarid regions, soil salinity is one of the major environmental constraints for crop production (1). The United Arab Emirates (UAE), located in the arid region, is often characterized by low rainfall and low-quality groundwater. In coastal regions, the overuse and decline of groundwater have caused seawater intrusion (3) and resulted in many farms becoming unproductive or totally abandoned (4). This has severely affected ruminant animal production, leading to a dire need to address feed shortages. In this context, where water is becoming a scarce commodity, shift to the cultivation of salt-tolerant plants with a high level of crude protein in these areas is a potential approach for the sustainability of the livestock industry.

Plants respond to salinity differently including delayed germination, high seedling mortality, poor crop stand, stunted growth, and reduced yield (5) because the increase in osmotic tension of soil solution reduces water absorption by roots and results in the accumulation of various ions in toxic levels (6). The unfavorable impact of saline water irrigation directly affects the soil-water-plant relations (7). Panicum is a well-known warm-season grass widely distributed in many regions. This grass has been extensively studied, with research focused on pasture establishment, production, and nutritive value throughout vegetative growth (8). The grass species *Panicum maximum* (Mombasa) presents one of the greatest potential production of dry matter in subtropical and tropical environments (9). *Panicum antidotale* (blue panic), an excellent sand binder that favors arid and semiarid conditions, is a salt-tolerant crop cultivated under moderate saline conditions (10). Blue panic is also considered an ideal fodder grass due to its high protein content and its ability to adapt to severe environmental stresses such as drought, salinity, and toxic nutrients (5, 11).

Although goats are worldwide providers of essential meat and dairy products, little research is performed on them (12). In order to survive adverse climatic conditions, animals have developed alterations in their renal and liver functions (13, 14). The biochemical and hematological constituents of blood are markers to determine the efficacy of the feed nutrient content and its utilization (15).

We hypothesized that the feeding of Mombasa or blue panic as a salt-tolerant alternative to alfalfa can affect the growth performance, blood hematology, serum metabolites, and blood mineral profile of growing goats kept under arid conditions. Since the biochemical and hematological parameters are considered a clear indication of animal wellness (2), the objective of this study was to evaluate the effects of feeding Mombasa or blue panic as a salt-tolerant alternative to Alfalfa on growth, hematological, and biochemical parameters of Ardi (Aardi or Ardhi) goats under Abu Dhabi conditions.

## Materials and methods

### Ethical approval

The authors confirm that the ethical policies of the journal, as noted on the journal's author guidelines page, have been adhered to, and the appropriate ethical review committee approval has been received. All procedures of the current study were followed according to Directive 2010/63/EU of the European Parliament and the Council of 22 September 2010 on the safety of animals used for scientific purposes.

### Description of the study site

The research was conducted at Baniyas Research Station, Abu Dhabi Agriculture and Food Safety Authority, Abu Dhabi, United Arab Emirates. The site represents a hot, arid climate, and the soil is sandy with chemical characteristics, as shown in Supplementary Table 1.

### Plant preparation and chemical composition

*Panicum maximum* cv. Mombasa and *Panicum antidotale* (blue panic) were cultivated at salinity levels ranging from 6,000 to 12,000 ppm.

Grasses were harvested at 28–42-day intervals between cuts throughout the 1-year cycle, mixed with the commercial concentrate feed (ruminant animal ration, National Feed and Flour Production and Marketing Co. L.L.C., Abu-Dhabi, UAE), and then directly presented to animals. Dry samples of alfalfa hay, Mombasa, blue panic, and commercial concentrate feed, which were used in the animal diet, were analyzed for crude protein (CP) according to (16). Acid detergent fiber (ADF) and neutral detergent fiber (NDF) were measured as described by (17). The proximate nutrient composition of commercial concentrate feed, Alfalfa hay, blue panic, and Mombasa, which were used in the animal diet, are presented in Table 1.

**Table 1:** Nutrient composition of Mombasa, blue panic, alfalfa, and commercial concentrate feed (*n* = 5/feed type).

**Feed type**		**Alfalfa**	**Mombasa**	**Blue panic**	**Concentrate**
Dry Matter%		92.49	91.15	91.47	90.76
ADF%		39.45	27.72	23.15	17.9
N.D. Fiber%		70.1	62.69	58.56	32.09
Crude Protein%		11.94	12.31	16.56	9.94
Macro Elements%	**N**	1.91	1.97	2.65	1.59
	**P**	0.26	0.38	0.35	0.37
	**k**	0.93	0.79	0.76	0.84
Secondary	**Ca**	0.55	0.81	0.68	1.44
Elements%	**Mg**	0.44	0.54	0.35	0.24
	**Na**	0.29	0.31	0.25	0.21
	**S**	0.63	1.72	0.64	0.41
Trace and Heavy	**Fe**	145.5	141.9	183	344
Metals ppm	**Cu**	21.5	15.6	20.7	40.7
	**Mn**	16.4	22.8	33.9	75.9
	**Zn**	26.7	46.1	68.4	65.2
	**Cr**	1.59	1.72	7.76	2.89
	**Se**	0	3.81	1.33	0.79

ADF, acid detergent fiber; P, phosphorus; K, potassium; Ca, calcium; Mg, magnesium; S, sulfur; Na, sodium; Fe, iron; Cu, copper; Mn, manganese; Zn, zinc; Cr, chromium; Se, selenium. The ingredients of the commercial concentrate feed were barley, yellow corn, soybean meal, wheat bran, sodium bicarbonate, molasses, lime stone, salt, monocalcium phosphate, mycotoxin binder, vitamins and mineral premixes, anticoccidials, and other essential feed additives.

### Animal management and treatment diets

Twenty-four growing female goats (120 ± 7 days) at an average weight of 14.45 ± 0.6 kg were used in this experiment. The goats had a clinically normal, healthy appearance after physical examination by a veterinarian (body temperature, grazing ability, ruminal activity, limbs and moving activities, and examination of the mouth and mucous membrane of eyes). The animals were housed outdoors with a shelter covering 60% of the farm during the day and in a semi-open barn at night, and they got clean water *ad libitum*. The goats were randomly allocated into three groups as follows: (1) the control group, which was fed alfalfa hay and concentrate, (2) Mombasa group, which was fed *Panicum maximum* cv. Mombasa and concentrate, and (3) blue panic group, which was fed *Panicum antidotale* and concentrate. All diets were calculated and formulated to contain CP and energy to meet the nutritional requirements for growing goats, according to (18). Feeding treatments were applied for 8 consecutive weeks after 4 weeks of adaption period.

### Growth performance and digestibility

The weight of an individual animal was measured at the onset of the trial and subsequently on a weekly basis until the end of the trial duration. The difference between the initial and final weights was used to compute weight change (gain/loss) for goats in each dietary treatment. During the last week of the experiment, the animals in each group (eight/group) were independently housed in metabolic cages for the digestibility experiment. Feces were collected from each animal, dried in the oven at 60°C for 24 h, and stored for laboratory analysis. The apparent coefficients of crude protein and acid detergent fiber digestibility were determined using a direct method (19).

### Puberty

Puberty age, the age at which a female goat exhibits its first heat (estrus), was recorded. Goats in heat exhibited all or some of the following signs: mucous discharge from the vulva, swollen vulva, bleating, frequent tail wagging, pacing down the fence line, and standing heat.

### Hematologic and serum biochemical analyses

At the end of the experiment, the goats were restrained for blood collection, and a pair of 7 ml blood samples were withdrawn into test tubes *via* a jugular vein puncture: one for hematological analysis and the other for serum chemistry. The sterile test tubes containing EDTA as an anticoagulant were sent to the laboratory within an hour of collection for the determination of hematological parameters. Blood tubes without anticoagulant were allowed to coagulate, centrifuged at 3,000 rpm at 21°C for 10 min, and stored at −20°C for biochemical analysis. The hematological estimates include hemoglobin (Hb), haematocrit (PCV), red blood cells (RBCs), white blood cells (WBCs), differential leucocytic count, mean corpuscular volume (MCV), mean corpuscular hemoglobin (MCH), and mean corpuscular hemoglobin concentration (MCHC) were analyzed by a Sysmex XT 2000i Analyzer (VLD-DPM-CBC-06, Mundelein, IL 60060, USA). The serum concentration of biochemical metabolites including creatinine (Crea), total protein (TP), albumin (ALB), urea (BUN), creatinine kinase (CK), alanine aminotransferase (ALT), aspartate aminotransferase (AST), low-density lipoprotein (LDL), alkaline phosphatase (ALP), and gamma-glutamyl transferase (GGT) was determined using commercially available kits (Shimadzu, Kyoto, Japan) according to the manufacturer's instructions using Beckman Coulter analyzers (VLD-DPM-CBC-02, Mundelein, IL 60060, USA). Sodium, potassium, chloride, iron, copper, zinc, phosphorus, and calcium serum concentrations were determined using the ion-selective electrode (ISE) module of the Roche/Hitachi cobas c systems (Model c501).

### Statistical analysis

Data were statistically analyzed with a one-way ANOVA using a statistical software program (SPSS for Windows, version 16.0, SPSS Inc., Chicago, IL). Duncan's *post hoc* test was applied to determine the level of significance between the groups. The data were presented as the mean ± standard error of mean (SEM). Results of statistical analysis were considered significant at *P* < 0.05.

## Results

### Growth performance

After a feeding trial of 12 consecutive weeks, changes in weight were determined, as shown in Table 2. There were no significant differences in the final body weight and total body weight gain between the treated groups (Table 2). Concerning acid detergent fiber, neutral detergent fiber, and crude protein digestibility, the goats fed blue panic had increased (*P* < 0.05) crude protein and neutral detergent fiber digestibility compared with Mombasa- and Alfalfa-fed groups (74.61 ± 0.49 vs. 69.26 ± 2.11 and 67.24 ± 0.81, respectively, for crude protein digestibility and 62.78 ± 0.46 vs. 58.39 ± 1.07 and 58.98 ± 0.78, respectively, for neutral detergent fiber digestibility). At the same time, no significant influence was recorded among different treatments for acid detergent fiber digestibility (Table 3).

**Table 2:** Growth performance in growing goats fed alfalfa, Mombasa, or blue panic.

	**Alfalfa**	**Mombasa**	**Blue panic**
**IBW (kg)**	14.4 ± 0.68	14.48 ± 0.73	14.46 ± 1.6
**FBW (kg)**	18.69 ± 0.77	17.88 ± 0.67	18.35 ± 1.58
**BWG (kg)**	4.29 ± 0.47	3.4 ± 0.34	3.89 ± 0.16

IBW, initial body weight; FBW, final body weight; BWG, body weight gain. Means in the same row with no superscript letters are not significantly different (p > 0.05).

**Table 3:** Digestibility of crude protein, acid detergent fiber, and neutral detergent fiber in growing goats fed alfalfa, Mombasa, or blue panic.

	**Alfalfa**	**Mombasa**	**Blue panic**
**CP (%)**	67.24 ± 0.81^b^	69.26 ± 2.11^b^	74.61 ± 0.49^a^
**ADF (% of DM)**	39.43 ± 0.56	38.15 ± 0.43	39.98 ± 0.74
**NDF%**	58.98 ± 0.78^b^	58.39 ± 1.07^b^	62.78 ± 0.46^a^

CP, crude protein; ADF, acid detergent fiber; NDF, neutral detergent fiber. Means in the same row with no superscript letters after them or with a common superscript letter following them are not significantly different (p > 0.05).

### Puberty

**Figure 1:**
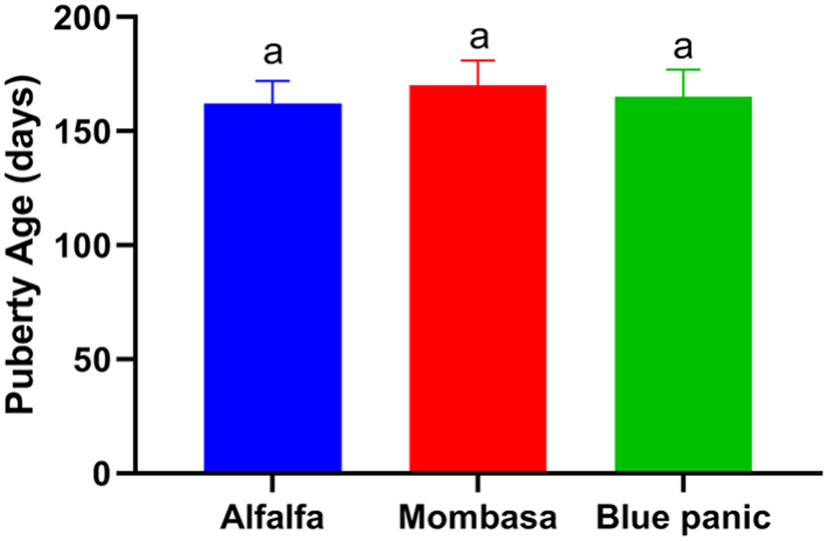
Puberty age in goats fed with alfalfa, Mombasa or blue panic.

The puberty age did not differ significantly between the goats fed on blue panic, Mombasa, and Alfalfa (Figure 1).

### Hematologic analysis

HCT (%), Hb (g/dl), RBCs (10^6^/μl), MCH (pg), MCHC (g/dl), and WBCs (10^3^/μl) were similar among the treatment groups (Table 4). The MCV was significantly (*P* < 0.05) higher in the alfalfa group than in the Mombasa and blue panic groups. Component WBCs were also evaluated, and the results are shown in Table 4. There were no treatment effects on lymphocytes (%), monocytes (%), neutrophils (%), basophils (%), and eosinophils (%) (*P* > 0.05).

**Table 4:** Blood hematology in growing goats fed alfalfa, Mombasa, or blue panic.

	**Alfalfa**	**Mombasa**	**Blue panic**
HCT (%)	35.45 ± 1.06	32.15 ± 0.67	32.3 ± 1.4
Hb (g/dl)	12.5 ± 0.26	12.2 ± 0.46	12.03 ± 0.41
RBCs (10^6^ /μl)	17.83 ± 0.43	17.58 ± 0.4	17.85 ± 0.61
MCV (fl)	19.91 ± 0.51^a^	18.31 ± 0.39^b^	18.05 ± 0.25^b^
MCH (pg)	7.03 ± 0.08	6.94 ± 0.2	6.74 ± 0.12
MCHC (g/dl)	35.36 ± 0.64	37.96 ± 1.25	37.44 ± 1.06
WBCs (10^3^ /μl)	14.38 ± 0.9	15.9 ± 2.68	13.68 ± 1.27
LYMPH (%)	44.7 ± 3.49	44.64 ± 4.87	53.9 ± 1.08
BASO (%)	2.84 ± 0.53	2.56 ± 0.26	2.31 ± 0.44
EOSINO (%)	4.51 ± 1.4	1.69 ± 0.38	2.26 ± 0.26
NEUT (%)	43.85 ± 3.48	45.25 ± 4.69	36.53 ± 1.15
MONO (%)	4.1 ± 1.03	5.86 ± 1.31	5 ± 0.67

HCT, hematocrit; HB, hemoglobin; RBCs, red blood cells; MCV, mean corpuscular volume; MCH, mean corpuscular hemoglobin; MCHC, mean corpuscular hemoglobin concentration; WBCs, white blood cells; LYMPH, lymphocytes; NEU, neutrophil; MONO, monocytes; ESOIN, eosinophil; BASO, basophils. Means in the same row with no superscript letters after them or with a common superscript letter following them are not significantly different (p > 0.05).

### Serum metabolites

The effects of feeding on serum metabolites are presented in Table 5. Creatinine (mg/dl), total protein (g/dl), albumin (g/dl), CK (IU/l), ALT (IU/l), AST (IU/l), LDH (IU/l), and GGT (IU/l) were similar among the treatments. However, glucose (mg/dl) levels were significantly (*P* < 0.05) increased in the blue panic-treated group compared with the Alfalfa- and Mombasa-treated groups (47.26 ± 3.88 vs. 36.18 ± 3.11 and 33.15 ± 2.35, respectively). Similarly, urea (mg/dl) values were increased (*P* < 0.05) in the Mombasa-treated group (21.66 ± 0.56) compared with the blue panic- and Alfalfa-treated groups (17.98 ± 1.05 and 17.83 ± 0.99, respectively).

**Table 5:** Serum metabolites in growing goats fed alfalfa, Mombasa, or blue panic.

	**Alfalfa**	**Mombasa**	**Blue Panic**
**Glucose (mg/dl)**	36.18 ± 3.11^b^	33.15 ± 2.35^b^	47.26 ± 3.88^a^
**BUN (mg/dl)**	17.83 ± 0.99^b^	21.66 ± 0.56^a^	17.98 ± 1.05^b^
**Crea (mg/dl)**	0.45 ± 0.02	0.48 ± 0.02	0.46 ± 0.05
**TP (g/dl)**	6.37 ± 0.14	6.48 ± 0.13	6.32 ± 0.1
**ALB (g/dl)**	3.41 ± 0.05	3.32 ± 0.03	3.46 ± 0.04
**CK (IU/l)**	367.25 ± 29.94	370.75 ± 22.5	318.38 ± 22.36
**ALT (IU/l)**	22.1 ± 1.95	22.9 ± 1.07	21.85 ± 0.7
**AST (IU/l)**	77.6 ± 3.23	78.98 ± 4.76	77.29 ± 3.33
**LDH (IU/l)**	432.25 ± 12.93	434.5 ± 20.65	387.38 ± 10.53
**GGT (IU/l)**	35.5 ± 3.77	42.63 ± 8.38	39.13 ± 1.16

Creatinine, Crea; TP, total protein; ALB, albumin; BUN, urea; CK, creatinine kinase; ALT, alanine aminotransferase; AST, aspartate aminotransferase; LDL, low-density lipoprotein; ALP, alkaline phosphatase; GGT, gamma-glutamyl transferase. Means in the same row with no superscript letters after them or with a common superscript letter following them are not significantly different (p > 0.05).

### Minerals

As shown in Table 6, phosphorus (P), sodium (Na), potassium (K), and chloride (Cl) were significantly influenced by the diet: whereas the blue panic group showed significantly (*P* < 0.05) increased P, Na, and Cl concentrations in serum compared with the other two groups, while the Mombasa group showed significantly (*P* < 0.05) increased K concentration in serum compared with the other groups. On the other hand, calcium (Ca), zinc (Zn), iron (Fe), and copper (Cu) levels were not statistically affected by feeding.

**Table 6:** Serum minerals in growing goats fed alfalfa, Mombasa, or blue panic.

	**Alfalfa**	**Mombasa**	**Blue Panic**
Ca	9.85 ± 0.14	9.56 ± 0.13	9.66 ± 0.11
P	10.74 ± 0.32^b^	11.85 ± 0.7^ab^	13.05 ± 0.63^a^
Fe	122.25 ± 4	123.48 ± 7.01	117.61 ± 6.73
Na	149.75 ± 0.41^c^	151.88 ± 0.48^b^	154.75 ± 0.59^a^
K	6.43 ± 0.17^b^	8.29 ± 0.37^a^	6.99 ± 0.33^b^
Cl	105.69 ± 0.47^c^	108.66 ± 0.74^b^	112.76 ± 0.7^a^
Zn	102 ± 17.59	106.88 ± 21.93	91 ± 6.52
Cu	131.75 ± 16.06	133.5 ± 19.57	115.63 ± 3.72

Means in the same row with no superscript letters after them or with a common superscript letter following them are not significantly different (p > 0.05).

## Discussion

Plants depend on different signaling cascades to tolerate different stresses, including soil salinity, but little is known about how plants deal with concurrent multiple stresses (20). In this study, we examined the resistance of Mombasa and blue panic cultivars under salinity stress conditions as salt-tolerant alternatives to Alfalfa and assessed their effects on the growth performance, blood hematology, serum metabolites, and blood mineral profile of growing goats kept under arid Abu Dhabi conditions. We observed good growth performance of the goats reared under tropical and subtropical conditions in all treatment groups, indicating that salt-tolerant Mombasa and blue panic could successively substitute Alfalfa in small ruminant diets.

The onset of puberty in sheep is believed to be influenced by diet, and lambs with faster growing rates are supposed to exhibit their first estrus at an earlier age. Most sheep breeds possess a marked seasonality of breeding activity (21). Surprisingly, lambs that fail to achieve puberty in their first autumn will be delayed until the next breeding season (22). Therefore, we started deciphering the possible effect of Mombasa or blue panic as a salt-tolerant alternative to Alfalfa on age at puberty of ewe lambs. In the present study, no diet effect on age at puberty was observed. This confirms the previous studies which reported that the age at puberty was not affected by diet in sheep fed on high-forage and high-grain diets (23), or in heifers fed on ionophore (24). This highlights the possibility of replacing Alfalfa in a small ruminant diet with salt-tolerant forages such as Mombasa or blue panic.

It is broadly recognized that biochemical and hematologic variables of blood can be considered for monitoring and or evaluating health conditions proficiently, and nutritional and physiological statuses of ruminants and for assisting in identifying various environmental stresses (25, 26). Concerning the present findings, blood hemoglobin (g/dl), hematocrit (%), RBC (10^6^/μl), WBC (10^3^/μl), and MCHC (g/dl) were not influenced by diet; however, these results were within normal ranges reported for clinically healthy goats (2, 27). The normal values reported in this study may be attributed to the absence of hemolytic and microcytic hypochromic anemia, which is associated with improper iron utilization during the formation of Hb (13). On the other hand, our results showed that the diet influenced the MCV, which was higher in the Alfalfa group. The fact that Alfalfa only resulted in higher MCV (fl) levels is in agreement with previous studies which reported that concentrate diets did not significantly influence most of the blood metabolites (28, 29). WBCs are essential for the immune system, particularly lymphocytes and monocytes which are precursors of macrophages are important for cell-mediated and humoral immunity responses (30). The results from the current study show no effects of fed on total and differential leukocyte counts, suggesting that the fed diets have no negative effects on the immune system, as was also reported by a previous study (30).

Serum biochemical indices were within the normal range in all groups, indicating the normal function of the kidney and liver (31–33). The serum concentration of glucose was higher in the blue panic-fed group, which may be attributed to better CP and neutral detergent fiber digestibility. It has been documented that blood urea could be used as an indirect indicator of the protein composition of feed as increased serum urea concentration is linked with higher ruminal degradation of the protein (34). Moreover, blood urea nitrogen was reported to increase when there is catabolism of body protein (35). The serum urea levels in the current study increased in the Mombasa-fed group. The possible reason for this increase could be the increase in DM intake exhibited by goats and/or higher ruminal degradation of the protein. A previous study showed that blood urea N increases in cases of rumen ungradable protein and where very large volumes of amino acids are consumed (36). The albumin content and liver enzymes were within the acceptable range, indicating no negative effects of diets on the liver (37).

Microelements have shown beneficial effects in maintaining cellular physiological functions (38) and enhancing the antioxidant defense mechanism of cells in addition to reducing the harmful effect of the hot ambient temperature (39, 40). The chemical composition of the plants used in the current study showed that Mombasa and blue panic have a high number of microelements such as Zn, Mn, and Se, which play vital roles as antioxidant agents during heat stress conditions (41). Additionally, the serum concentrations of P, Na, and Cl increased in the blue panic-fed group, while the Mombasa group showed an increase in the K concentration, suggesting that Mombasa and blue panic can support the same level of growth as alfalfa.

## Conclusion

It could be concluded that feeding Mombasa or blue panic as a salt-tolerant alternative to replacing Alfalfa had no adverse effects on growth performance, puberty, feed utilization, serum metabolites, and serum minerals in growing goats reared under arid conditions in Abu Dhabi. This study provides new insights into the shift to the cultivation of salt-tolerant plants with a high level of crude protein in arid areas, which is a potential approach for the sustainability of the livestock industry. Further studies are needed for the assessment of sustainability after using salt-tolerant alternative food sources to traditional Alfalfa.

## Data availability statement

The original contributions presented in the study are included in the article/Supplementary material, further inquiries can be directed to the corresponding authors.

## Ethics statement

The animal study was reviewed and approved by the Directive 2010/63/EU of the European Parliament and the Council of 22 September 2010, on the safety of animals used for scientific purposes.

## Author contributions

HZ and IA-A conceived, designed, and performed the experiments. AM analyzed the data and wrote the manuscript. AA, AT, and AS contributed to the analysis tools. HZ and AM revised and edited the manuscript. All authors contributed to the article and approved the submitted version.

## Funding

This research was funded by the Abu Dhabi Agriculture and Food Safety Authority.

## Conflict of interest

The authors declare that the research was conducted in the absence of any commercial or financial relationships that could be construed as a potential conflict of interest.

## Publisher's note

All claims expressed in this article are solely those of the authors and do not necessarily represent those of their affiliated organizations, or those of the publisher, the editors and the reviewers. Any product that may be evaluated in this article, or claim that may be made by its manufacturer, is not guaranteed or endorsed by the publisher.
