# Supplementary material for: Hematological and biochemical indices, growth performance, and puberty of goats fed with Mombasa and blue panic as salt-tolerant alternatives to alfalfa under arid conditions
Source: Front Vet Sci. 2022 Oct 18;9:961583. doi: 10.3389/fvets.2022.961583 (PMC9622799; doi:10.3389/fvets.2022.961583)
Supplement: Supplementary file 1 [file Table_1.DOCX]

Supplementary Material

# Supplementary Tables

**Supplementary Table 1.** Chemical properties of the experimental soil.

| **Soil depth (cm)** | **pH** | **EC_e_ (dSm^-1^)** | **CaCO_3_ (%)** | **Total N (ppm)** | **Available P (ppm)** | **Exchangeable K (ppm)** |
| --- | --- | --- | --- | --- | --- | --- |
| 20 | 7.95 | 1.8 | 29.8 | 535 | 146 | 101 |
